# Abdominal pain with a twist

**DOI:** 10.1186/1865-1380-4-21

**Published:** 2011-06-02

**Authors:** Rachael Mathews, Sam Thenabadu, Thiagarajan Jaiganesh

**Affiliations:** 1 University Hospital of Lewisham-London, London, UK; 2 Emergency Department, St Georges Hospital-London, Blackshaw Road, Tooting, London, SW17 0QT, UK

## Abstract

Malrotation in children is due to either an incomplete or non-rotation of the foetal mid-gut during perinatal development. Presentation is usually in the first few weeks of life, often with life-threatening volvulus and ischaemia. However, it can be a rare cause of abdominal pain in older children and young adults. We present such a case, as a reminder to emergency physicians that malrotation should be considered in the differential diagnosis of recurrent or chronic abdominal pain not only in children but also in adolescents.

## The Case

A 14-year-old boy presented to the paediatric emergency department (PED) with a 24-h history of intermittent right-sided abdominal pain and bilious (greenish) vomiting that had settled just prior to his arrival to the PED. His haemodynamic parameters were normal and clinical examination including an abdominal examination was unremarkable.

His past medical history revealed that he had experienced at least four identical previous episodes of abdominal pain with vomiting that necessitated attending three different emergency departments over a 1-year period. On each occasion he had been admitted to a general paediatric or a paediatric surgical ward with provisional diagnoses of evolving appendicitis, but was discharged home as his blood results including inflammatory markers and abdominal ultrasound scans were normal. He had received quadruple therapy (bismuth salts, amoxicillin, omeprazole and metronidazole) for *H. pylori *infection identified on stool antigen test and on hydrogen breath test almost 6 months prior to this presentation. He was awaiting an upper GI endoscopy because of his recurrent symptoms.

**Figure 1:**
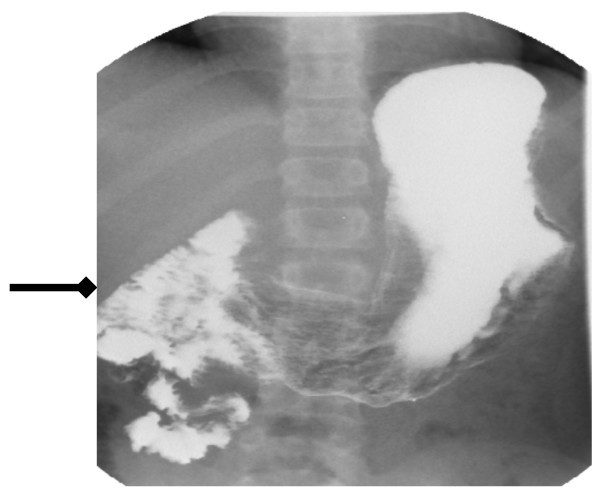
**Duodenum and the small bowel lying on the *right side *of the midline (*arrow*)**.

He was admitted to the paediatric ward on this presentation for evaluation of this recurrent abdominal pain and concerning bilious vomiting. He subsequently underwent an upper GI contrast study as an inpatient. The x-ray (Figure 1) findings were that the stomach was normal and that there was no small bowel hold up. It was noticed that the duodenum and the entire small bowel was on the right side of the abdomen. A diagnosis of malrotation was made, and the patient was referred to the paediatric surgical team the same day. He underwent a successful Ladd's procedure.

## Discussion

Malrotation is the term used to define a spectrum of developmental abnormalities resulting in incomplete or nonrotation of the mid-gut. During foetal development between the 4th and 12th week of gestation, the mid-gut rotates anticlockwise around the axis of the superior mesenteric artery before assuming its final place in the abdomen and is fixed with the posterior abdominal wall. First described by Ladd in the early 20th century [1], failure of this process can have disastrous consequences, causing duodenal obstruction and intestinal ischaemia either due to a volvulus because of the narrow-based gut mesentery and failure of fixation, or occasionally due to the presence of 'Ladd's' bands (stalk of peritoneal tissue that attaches the caecum to the abdominal wall).

The incidence of malrotation is reported at 1 in 500 [2], with more than 60% of cases of malrotation presenting within the first week of life and around 85% by 1 year of age [3]. Thereafter, it can present in any age, including the elderly. There is a slight male preponderance of 2:1 until the first year of life and thereafter the ratio becomes equal [4].

Up to 70% of cases are associated with other congenital abnormalities, such as gastroschisis, omphalocele, intestinal atresias, anorectal malformations and cardiac/hepatic abnormalities. Malrotation is rarely seen in older children, and when it does occur, symptoms may be absent or intermittent.

The clinical presentation depends on the age of presentation and the location of the defect. Infants most frequently present with bile-stained emesis. Pain and irritability are not prominent clinical features, and the abdomen is usually soft and non-tender unless progression to ischaemia has occurred. At this point, the abdomen becomes distended and tender, and the emesis may be blood stained.

Presentation later in life is vaguer, as our case illustrates. Making a diagnosis is often more difficult, because there is a larger spectrum of differentials to consider including annular pancreas, intussusceptions, duodenal web, etc. Symptoms often include abdominal pain and vomiting, although in a large proportion of case, the emesis is non-bile-stained. Gastro-oesophageal reflux, anaemia secondary to occult bleeding [5], malnutrition and even immunodeficiency have been described as possible presentations [6]. Disturbance of bowel habits such as chronic diarrhoea is also present, and can lead to confusion and delay the diagnosis. As shown in our case, recurrent pain due to intermittent volvulus is not uncommon.

Plain abdominal x-ray is normal in a simple malrotation. If there is a mid-gut volvulus then the classic radiographic finding is that of a double-bubble appearance where the first bubble is due to gastric dilatation and the second bubble is due to the dilatation of the first part of the duodenum. Abdominal ultrasound/CT scan [7] can be a useful adjunct as it identifies the position of the superior mesenteric vessels, but, as our case confirms, cannot be solely used to rule out a malrotation because of their low specificity. A colour Doppler may aid the diagnosis by producing a characteristic whirlpool-type blood flow in the superior mesenteric vein [8]. Although a barium enema can be used to detect an abnormal position of the caecum, the caecum can be sited normally in up to 20% of patients. The investigation of choice is an upper gastrointestinal contrast study [9], which reveals either an obstruction or the infamous corkscrew appearance of the duodenal-jejunal flexure not crossing the midline.

Initial management in an emergency presentation would involve fluid resuscitation, NG tube placement ('drip and suck'), correction of electrolyte abnormalities (hyponatraemia and hyperkalaemia) and administration of broad-spectrum intravenous antibiotics. Surgery is the treatment of choice as there is a high risk of vascular compromise and intestinal necrosis. A classic Ladd procedure is described as a reduction of the volvulus (if present), division of mesenteric bands, placement of small bowel on the right and large bowel on the left of the abdomen, and appendicectomy. An appendicectomy is carried out, partly because blood supply to the appendiceal vessels can be compromised, but also to prevent future diagnostic confusion as the appendix would lie in the left upper quadrant of the abdomen alongside the repositioned caecum. Ladd's operation is usually an open procedure; however, a modified laparoscopic technique has also been described [10,11].

Whilst mortality following Ladd's procedure remains low at 2%, this figure appears to be higher in those with intestinal ischaemia and even higher in the presence of intestinal necrosis/perforation and those with other co-morbidities [12]. Morbidity is also highest in these groups, particularly because of development of short-gut syndrome. Prophylactic Ladd's procedure is carried out even in asymptomatic malrotation particularly in children without any comorbidities because of the devastating consequences of a mid-gut volvulus and the quick recovery in this age group. This approach is not evidence based in the adolescent and adult subjects. However, it was carried out in our patient as it was thought that the recurrent pain was due to an intermittent volvulus.

## Conclusion

Malrotation can present even in the adolescent age group, and emergency physicians must be aware of this condition. Recurrent/chronic episodes of abdominal pain with bilious vomiting must be thoroughly investigated and less common differentials considered.

## Patient consent

Written informed consent was obtained from the patient for publication of this case report and any accompanying images. A copy of the written consent is available for review by the Editor-in-Chief of this journal.

## Competing interests

The authors declare that they have no competing interests.

## Authors' contributions

RM wrote the first draft of the paper. ST co-authored the first draft and reviewed all drafts and radiographic images. TJ reviewed and commented on all the drafts of the paper and radiographic images. All authors read and approved the final manuscript.
